# Grey matter atrophy in prodromal stage of dementia with Lewy bodies and Alzheimer’s disease

**DOI:** 10.1186/s13195-016-0198-6

**Published:** 2016-07-20

**Authors:** Frederic Blanc, Sean J. Colloby, Benjamin Cretin, Paulo Loureiro de Sousa, Catherine Demuynck, John T. O’Brien, Catherine Martin-Hunyadi, Ian McKeith, Nathalie Philippi, John-Paul Taylor

**Affiliations:** 1 Geriatrics day hospital and neuropsychology unit. Geriatrics department and Neurology service, Memory Resources and Research Centre (CMRR), University Hospital of Strasbourg, Strasbourg, France; 2 Team IMIS/Neurocrypto, French National Center for Scientific Research (CNRS), ICube Laboratory and Fédération de Médecine Translationnelle de Strasbourg (FMTS), University of Strasbourg, Strasbourg, France; 3 Institute of Neuroscience, Campus for Aging and Vitality, Newcastle University, Newcastle upon Tyne, UK; 4 Department of Psychiatry, University of Cambridge, Cambridge Biomedical Campus, Cambridge, UK

**Keywords:** Prodromal dementia with Lewy bodies, Dementia with Lewy bodies, Alzheimer’s disease, Alzheimer’s dementia, Prodromal Alzheimer’s disease, Lewy body disease, Mild cognitive impairment, MRI, Insula

## Abstract

**Background:**

Little is known about the patterns of brain atrophy in prodromal dementia with Lewy bodies (pro-DLB).

**Methods:**

In this study, we used SPM8 with diffeomorphic anatomical registration through exponentiated lie algebra to measure grey matter (GM) volume and investigate patterns of GM atrophy in pro-DLB (*n* = 28) and prodromal Alzheimer’s disease (pro-AD) (*n* = 27) and compared and contrasted them with those in elderly control subjects (*n* = 33) (*P* ≤ 0.05 corrected for family-wise error).

**Results:**

Patients with pro-DLB showed diminished GM volumes of bilateral insulae and right anterior cingulate cortex compared with control subjects. Comparison of GM volume between patients with pro-AD and control subjects showed a more extensive pattern, with volume reductions in temporal (hippocampi and superior and middle gyri), parietal and frontal structures in the former. Direct comparison of prodromal groups suggested that more atrophy was evident in the parietal lobes of patients with pro-AD than patients with pro-DLB. In patients with pro-DLB, we found that visual hallucinations were associated with relative atrophy of the left cuneus.

**Conclusions:**

Atrophy in pro-DLB involves the insulae and anterior cingulate cortex, regions rich in von Economo neurons, which we speculate may contribute to the early clinical phenotype of pro-DLB.

## Background

Dementia with Lewy bodies (DLB) is the second most common form of dementia after Alzheimer’s disease (AD), accounting for 15–20 % of neuropathologically defined cases [1]. Diagnostic classification of DLB is based on revised consensus criteria, with core diagnostic features of DLB being (1) recurrent visual hallucinations, (2) cognitive fluctuations and (3) spontaneous motor features of parkinsonism [1]. The presence of two or three of these core signs is sufficient for a diagnosis of probable DLB [1] at the stage of dementia, and this overlaps with the recent nomenclature update in the *Diagnostic and Statistical Manual of Mental Disorders, Fifth Edition* (DSM-V), where probable DLB is now comes under the heading of ‘major neurocognitive disorder with Lewy bodies’ [2]. In addition, DSM-V has the diagnostic classification of mild neurocognitive disorder with Lewy bodies. Thus, there is formal recognition that there is a pre-dementia state of this disease [2], similar to AD, where there is underlying neurodegenerative disease but function has not yet been compromised. This state has also been referred to as *prodromal DLB* (pro-DLB), a term which we use in this paper.

Distinguishing DLB from AD at the dementia stage is difficult because of overlapping clinical and neuropathological features between the two conditions, as well as because specific symptoms of DLB, such as hallucinations or fluctuations, are not spontaneously described by the patient and the caregiver. At early or prodromal stages, the challenges are even greater, given the subtlety of symptoms present. Whilst cognitive patterns in pro-DLB have been described as different from prodromal AD (pro-AD) [3, 4], with patients with DLB at this early stage having more visuospatial and fluency deficits than those with AD, and patients with AD having more amnesic impairments than those with DLB [4, 5], the neuropsychological pattern of pro-DLB appears to be more heterogeneous than that in pro-AD [5]. However, verbal memory [6] and visual memory impairments are frequent at the early stage of DLB, as in AD [7], and that is the reason why the differentiation of AD and DLB is difficult at the individual level. The behaviour modifications are also common in the two diseases: anxiety and depressive symptoms are frequent and do not permit differentiate of the two diseases [8, 9].

However, accurate differentiation of DLB and AD, regardless of the stage of the disease, is important clinically, given different management trajectories for each disease (e.g., avoidance of neuroleptics in DLB, but likely better response to cholinesterase inhibitors [10]) as well as prognosis [11]. Furthermore, accurate early subtype diagnosis of the underlying neurodegenerative cause is becoming increasingly important for ensuring that future disease-modifying treatments can be targeted in individuals before substantive neurodegenerative deficits have occurred. Similarly, identification and validation of early biomarkers of pro-DLB will further assist in the development of pro-DLB criteria [12]. Specifically, for purposes of our analyses, and in alignment with our previous work [13], we consider individuals with pro-DLB to be those patients who meet the revised diagnostic criteria for DLB, but, instead of dementia [1], fit the criteria for mild cognitive impairment (MCI) [14].

Structural neuroimaging represents one potential biomarker area, and a well-established method is the use of voxel-based morphometry (VBM) to study atrophic change in dementia. Comparison of VBM and a manual method for hippocampal volumetry to detect temporal lobe atrophy in AD showed that VBM with or without diffeomorphic anatomical registration through exponentiated lie algebra (DARTEL) registration is equivalent or more accurate [15, 16]. But VBM-DARTEL seems to be more accurate at detecting hippocampal atrophy in subtle cases, such as in depression [17]. This image-processing technique is also useful in many others neurological or psychiatric diseases including depression, schizophrenia, temporal lobe epilepsy, [18] but also MCI Parkinson’s disease [19].

Previous VBM studies in DLB at the stage of dementia have demonstrated diminished volume in the insulae bilaterally and in the lateral temporal lobes, frontal lobe and precuneus [20], and others have reported grey matter (GM) volumetric reductions in the mid-brain, the substantia innominata, the hypothalamus and the right insula [21], and these findings are confirmed by a voxel-wise meta-analysis on cortical atrophy of patients with DLB at the stage of dementia whose authors found bilateral insulae and basal ganglia atrophy [22]. Furthermore, this resonates with recent findings from our group. We demonstrated that the cortical thickness of patients with pro-DLB is diminished in the right anterior part of the insula [13]. This novel finding is significant, since patients at the early stage of the disease frequently have neurovegetative symptoms such as constipation, orthostatic dizziness or increased saliva, symptoms domains [23] which have been linked to insula. At the early stage, patients also frequently have cognitive fluctuations and hallucinations, and insular dysfunction has also been implicated in these symptoms [24, 25]. Contrarily, given that parkinsonism is rarely obvious at the beginning of the DLB, one might expect the putamen, pallidum and substantia nigra regions would be less structurally affected earlier in the disease [26]. However, data on subcortical structure were previously unexplored, since cortical in thickness studies researchers analysed only the cortex and not the hippocampi or basal ganglia [13].

Therefore, the primary aim of this study was to investigate cortical and subcortical grey matter (GM) atrophy patterns in patients with pro-DLB, and we report magnetic resonance imaging (MRI) patterns of GM atrophy in subjects with DLB at the stage of MCI (pro-DLB) and AD at the stage of MCI (pro-AD) compared with healthy elderly control subjects (HC). We hypothesised that, in pro-AD, the pattern of GM atrophy would involve predominantly the temporal lobe and parietal association cortices in keeping with the significant body of evidence supporting volumetric losses in these areas in pro-AD [27]. In contrast, we expected that the pattern of GM atrophy in pro-DLB would be less marked and more specific to structures such as the insulae.

## Methods

### Subjects, assessments and diagnosis

**Fig. 1:**
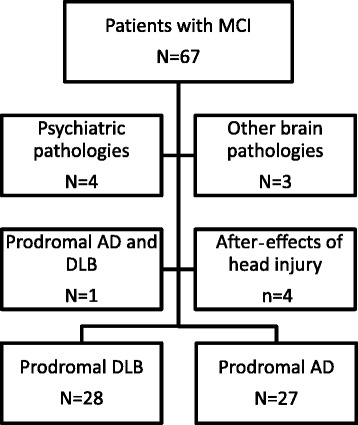
Flowchart of the present study on grey matter atrophy in prodromal stages of dementia with Lewy bodies and Alzheimer’s disease. Prodromal DLB is defined as patients with the McKeith et al. criteria of DLB with cognitive impairment but without dementia. Psychiatric pathologies included two patients with depression, one with bipolar disorder and one with histrionic personality disorder. In addition, among patients with MCI, one had cognitive impairment due to severe sleep apnoea, one had vitamin B_12_ encephalopathy and one had mitochondriopathy. *AD* Alzheimer’s disease, *DLB* dementia with Lewy bodies, *MCI* mild cognitive impairment

One hundred individuals suspected of having DLB or AD and HC over the age of 50 years were recruited (see Fig. 1 flowchart) from two European centres. Thirty-six were recruited from a community-dwelling population of patients referred to local old age psychiatry, geriatric medicine or neurology services in Newcastle upon Tyne (NCL); 64 were recruited from the tertiary memory clinic of Strasbourg (SXB), including neurology and geriatric medicine services. HC were recruited from established case registers. These subjects were previously included in a study of cortical thickness [13]. Subjects underwent detailed clinical and neuropsychological evaluations. Common elements between centres included the assessment of motor parkinsonism with the Unified Parkinson’s Disease Rating Scale Part III (UPDRS-III) [28], the Clinician Assessment of Fluctuation (CAF) [29], the Mini Mental State Examination (MMSE), the Clinical Dementia Rating scale (CDR) and the Trail Making Task A and B (TMTA and TMTB, respectively). For TMTA and TMTB, normative data from Tombaugh were used [30]. The neuropsychological evaluation of SXB included the Free and Cued Selective Reminding Tests for verbal memory, 48-item delayed matching to sample for visual recognition memory, forward and backward digit span, Wechsler Adult Intelligence Scale code for attention and speed processing, Frontal Assessment Battery and phonemic fluency for executive function, semantic fluency, oral denomination 80 items for language, the Rey-Osterrieth Complex Figure Test, and Mahieux praxis evaluation. The neuropsychological evaluation done at NCL was a comprehensive neuropsychological battery: the Cambridge Cognitive Examination as well as the F-A-S test and semantic fluency. For the purposes of this paper, we report only those scales which were common to both centres (e.g., MMSE, TMTA and TMTB).

Patients in the SXB group also underwent cerebrospinal fluid (CSF) analysis, including measurement of tau, phospho-tau, and amyloid-β_1–42_ (Aβ_1–42_; cut-off<500 ng/L) (INNOTEST® enzyme-linked immunosorbent assay; Fujirebio Europe, Gent, Belgium). Visual assessment of medial temporal atrophy on brain MRI scans was performed using the standardised Scheltens scale (five categories, range 0–4), with 0 corresponding to no atrophy [31]. The diagnosis of neurocognitive disorder for each patient was made using the Dubois et al. criteria for pro-AD (*n* = 27) [32]. Patients with pro-DLB (*n* = 28) were defined as patients with MCI (Petersen criteria) [33] and a CDR of 0 or 0.5, and also by the McKeith et al. criteria (meeting probable DLB criteria except presence of dementia) [1], which is consistent with recent suggestions regarding pro-DLB criteria [12]. Similarly, 33 aged healthy and cognitively intact (no MCI) subjects were recruited from among relatives and friends of subjects with neurocognitive disorders and individuals who volunteered via advertisements in local community newsletters in NCL and SXB areas. Exclusion criteria for participation in the study included contraindications for MRI, history of alcohol/substance misuse, evidence suggesting alternative neurological or psychiatric explanations for symptoms/cognitive impairment, focal brain lesions seen on brain imaging or the presence of other severe or unstable medical illness. All patients had formal assessment of their diagnosis done by independent expert clinicians (JPT and FB for NCL; and FB, BC and NP for SXB), and HC underwent clinical and cognitive assessments similar to those of patients to exclude any who may have had an occult MCI or dementia. Patients with concomitant AD and DLB (i.e., those meeting both McKeith et al. [for probable DLB] and Dubois et al. criteria) were excluded (see Fig. 1).

### MRI data acquisition

Subjects in the NCL and SXB groups underwent T1-weighted MRI on a 3-T MRI system within 2 months of the study assessment. NCL investigators used an 8-channel head coil (Intera Achieva scanner; Philips Medical Systems, Eindhoven, The Netherlands), and SXB researchers used a 32-channel head coil (Siemens Magnetom Verio syngo MR B17; Siemens Medical Solutions, Malvern, PA, USA). The sequence obtained was a standard T1-weighted volumetric sequence covering the whole brain (3D magnetization-prepared rapid gradient echo, sagittal acquisition, 1-mm isotropic resolution). Three-dimensional (3D) T1-weighted images of the NCL group had a matrix size of 240 (anterior-posterior) × 240 (superior-inferior) × 180 (right-left), a repetition time (TR) of 9.6 milliseconds, an echo time (TE) of 4.6 milliseconds and a flip angle of 8 degrees. 3D T1-weighted images of the SXB group had a matrix size of 192 (anterior-posterior) × 192 (superior-inferior) × 176 (right-left), a TR of 1900 milliseconds, a TE of 2.53 milliseconds and a flip angle of 9 degrees. The acquired volume was angulated such that the axial slice orientation was standardised to align with the anterior commissure-posterior commissure line.

### VBM-DARTEL

Analysis was conducted using the SPM8 software package (http://www.fil.ion.ucl.ac.uk/spm) running on MATLAB 7.9 (MathWorks, Natick, MA, USA). First, MRI scans were segmented into GM, white matter (WM) and CSF using SPM8’s standard unified segmentation module [34]. Second, using the DARTEL technique [35], a GM population template was derived from the entire imaging dataset (HC, pro-DLB and pro-AD). Third, after an initial affine transformation of the GM DARTEL template to the GM tissue probability map in Montreal Neurological Institute (MNI) space (http://www.mni.mcgill.ca/), non-linear warping of the segmented images was performed, matching the MNI space GM DARTEL template. Fourth, images were modulated to ensure that relative volumes of GM were preserved following spatial normalisation. Last, images were smoothed with an 8-mm full-width at half-maximum 3D Gaussian kernel. The spatially pre-processed scans were then used for voxel-wise statistical analysis.

### Statistical analysis

Controlling for age, total intracranial volume (TIV) and sex, group differences were assessed using the generalised linear model in SPM8, and statistical significance was estimated with the distributional approximations of Gaussian random fields [36]. Multiple regression analyses were also performed to investigate effects of GM loss on selected cognitive variables in pro-DLB (adjusted for age and TIV). Significant effects were identified using the corrected family-wise error (FWE) *P* value threshold (*P*_FWE_ ≤ 0.05). IBM SPSS version 22.0.0.0 software (IBM, Armonk, NY, USA) was used for further statistical evaluation as required. Where appropriate, differences in demographic and clinical data were assessed using parametric (analysis of variance [ANOVA], *t* test) and non-parametric (Kruskal-Wallis H test, Mann-Whitney *U* test) tests. In post hoc analyses, we employed Tukey’s test and the Mann-Whitney *U* test for ANOVA and Kruskal-Wallis tests, respectively. For categorical measures, χ^2^ tests were applied. For each test statistic, a probability value <0.05 was regarded as significant.

## Results

### Subject characteristics

The demographic data for patients and HC are summarised in Table 1. Subject groups were well matched for education, sex and handedness. Of the patients with pro-AD, 9 presented with an amnestic MCI single domain and 18 with amnestic MCI multiple domains. Of the patients with pro-DLB, 2 presented with amnestic MCI multiple domains, 13 with a non-amnestic MCI single domain and 13 with non-amnestic MCI multiple domains. For pro-AD and pro-DLB, MMSE and CDR scores were similar. For TMTA and TMTB, patients with pro-DLB were more impaired than the HC group. Patients with pro-DLB were more likely to have hallucinations and had higher motor parkinsonism (UPDRS III scores) and CAF scores. They also had a higher prevalence of rapid eye movement sleep behaviour disorder (RBD) than the prevalence in the other groups. Patients with pro-DLB were on dopaminergic treatment and had the highest use of neuroleptics (clozapine and quetiapine) compared with other groups. Patients with pro-AD had a greater number of abnormal CSF biomarkers than patients with pro-DLB. Among 23 patients with pro-DLB, only one had an abnormal level Aβ_1–42_. Upon visual rating of hippocampal atrophy on MRI scans, we found that patients with pro-AD had more atrophy than HC. Patients with pro-DLB had more atrophy than HC only for the left hippocampus. Patients with pro-AD had more hippocampal atrophy than patients with pro-DLB only for the right hippocampus.

**Table 1:** Clinical and demographic features of patients with dementia with Lewy bodies patients, Alzheimer’s disease at the mild cognitive impairment or prodromal stage, and healthy elderly control subjects

	Pro-DLB (*n* = 28; 2 NCL, 26 SXB)	Pro-AD (*n* = 27; 1 NCL, 26 SXB)	HC (*n* = 33; 30 NCL, 3 SXB)	Test statistic, *P* value	Post hoc analysis^a^
Age, years, mean (SD)	67.5 (9.2)	69.3 (7.8)	72.4 (10.4)	F = 2.189, *P* = 0.118	
Education,^b^ 1/2/3	11/4/8	11/3/12	1/14/11	H = 2.116, *P* = 0.347	
Sex, F/M	16/12	7/20	18/15	χ^2^ = 6.726, *P* = 0.035^c^	
Handedness, R/L	26/2	24/3	29/4	χ^2^ = 1.558, *P* = 0.459	
MMSE score	27.6 (2.1)	26.9 (1.9)	29.4 (0.9)	H = 31.897, *P* < 0.0001^c^	HC > pro-AD and pro-DLB
TMTA,^d^ impaired subjects	60.7 %	32.0 %	0 %	H = 12.174, *P* < 0.002^c^	HC > pro-DLB
TMTB,^d^ impaired subjects	71.4 %	44.0 %	0 %	H = 15.245, *P* < 0.0001^c^	HC > pro-DLB
CDR Sum of Boxes, 0/0.5/1/2/3	2/26/0/0/0	1/26/0/0/0	33/0/0/0/0	H = 75.466, *P* < 0.0001^c^	HC < pro-DLB and pro-AD
Parkinsonism^e^					
Rigidity, 0/1/2/3/4	7/20/1/0/0	23/4/0/0/0	33/0/0/0/0	H = 44.388, *P* < 0.0001^c^	Pro-DLB > HC and pro-AD
Akinesia, 0/1/2/3/4	10/14/3/1/0	23/4/0/0/0	31/2/0/0/0	H = 29.156, *P* < 0.0001^c^	Pro-DLB > HC and pro-AD
Tremor at rest, 0/1/2/3/4	17/9/2/0/0	27/0/0/0/0	33/0/0/0/0	H = 19.360, *P* < 0.0001^c^	Pro-DLB > HC and pro-AD
Hallucinations	60.7 %	0 %	0 %	χ^2^ = 44.521, *P* < 0.0001^c^	
Fluctuations	92.9 %	0 %	0 %	χ^2^ = 65.972, *P* < 0.0001^c^	
CAF^b^	3.5 (3.6)	0.0 (0)	0 (0)	H = 34.872, *P* < 0.0001^c^	Pro-DLB > HC and pro-AD
RBD	56.0 %	7.7 %	0 %	H = 31.696, *P* < 0.0001^c^	Pro-DLB > HC and pro-AD
Treatment					
ChEI	28.6 %	48.1 %	0.0 %	χ^2^ = 18.253, *P* < 0.0001^c^	
Dopa	28.6 %	0.0 %	0.0 %	χ^2^ = 19.274, *P* < 0.0001^c^	
NL	10.7 %	0.0 %	0.0 %	χ^2^ = 6.793, *P* = 0.033^c^	
CSF, mean (SD), number of subjects					
Aβ_1–42_	859.3 (336.7) *n* = 23	579.0 (287.4), *n* = 23	–	F = 5.345, *P* = 0.008^c^	Pro-DLB > pro-AD
Phospho-tau	43.6 (13.9), *n* = 23	93.7 (36.8), *n* = 23	–	F = 18.805, *P* < 0.0001^c^	Pro-DLB < pro-AD
Tau	313.0 (286.3), *n* = 23	660.6 (355.4), *n* = 23	–	F = 6.895, *P* = 0.002^c^	Pro-DLB < pro-AD
Hippocampal atrophy,^f^ 0/1/2/3/4					
Left hippocampus	14/10/2/2/0	5/16/4/2/0	27/5/1/0/0	H = 23.992, *P* < 0.0001^c^	HC < pro-AD and pro-DLB
Right Hippocampus	14/10/4/0/0	5/14/7/1/0	22/8/2/1/0	H = 13.591, *P* < 0.001^c^	Pro-AD < HC and pro-DLB

*Abbreviations: Aβ*_*1–42*_ amyloid-β_1–42_, *CAF* Clinician Assessment of Fluctuation, *CDR* Clinical Dementia Rating, *ChEI* cholinesterase inhibitor, *CSF* cerebrospinal fluid, *Dopa* levodopa or dopaminergic agonists, *HC* healthy elderly control subjects, *MMSE* Mini Mental State Examination, *NCL* old age psychiatry, geriatric medicine or neurology services from Newcastle upon Tyne, NL neuroleptic, *pro-AD* prodromal Alzheimer’s disease, *pro-DLB* prodromal dementia with Lewy bodies, *RBD* rapid eye movement sleep behaviour disorder, *SXB* tertiary memory clinic of Strasbourg, *TMT* Trail Making Test

^a^Tukey’s post hoc test for analysis of variance (F), Mann-Whitney post hoc test in IBM SPSS software (H)

^b^Education level: 1 = before high school, 2 = high school, 3 = university

^c^Statistically significant value

^d^percentage of patients with test failure according to the normative data of Tombaugh, 2004 [30]

^e^As rated on Unified Parkinson’s Disease Rating Scale [28]

^f^According to Scheltens et al. [31]

### Voxel-based morphometry group effects

**Fig. 2:**
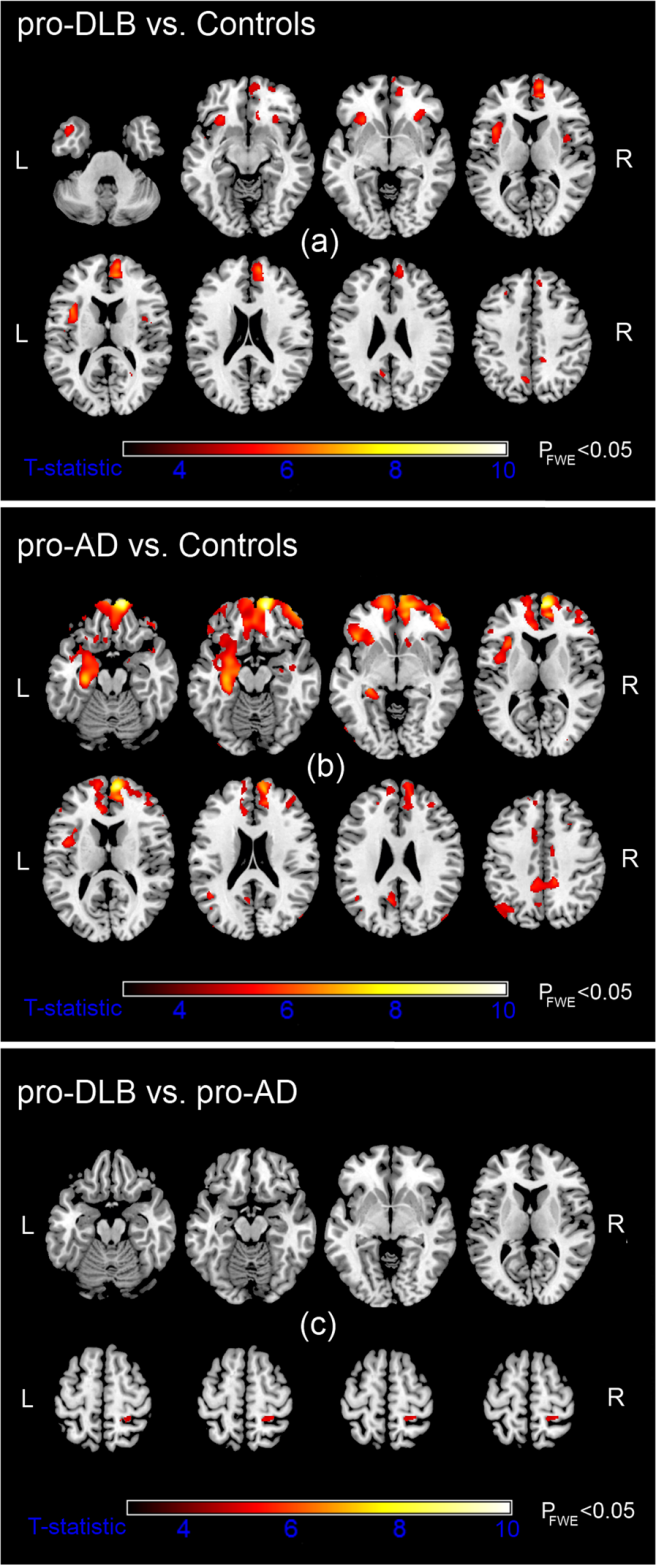
Significant grey matter (GM) loss in the prodromal dementia with Lewy bodies (pro-DLB) (**a**) and prodromal Alzheimer’s disease (pro-AD) (**b**) groups relative to healthy older control subjects. GM atrophy in pro-AD compared with pro-DLB (**c**). Results (*P*_FWE_ ≤ 0.05) are superimposed upon a magnetic resonance imaging T1-weighted brain template image in axial views. *L* left, *R* right, *FWE* family-wise error

VBM analysis revealed, relative to HC, significant GM volume loss in pro-DLB bilaterally in the insula, precuneus and medial frontal structures, as well as in the left anterior cingulate, left middle frontal, right superior and inferior frontal regions (Fig. 2a). Table 2 depicts the location and peak significance of these areas. No GM volume losses were apparent in HC that were greater than those in pro-DLB.

**Table 2:** Location and peak significance of grey matter volume loss using VBM-DARTEL

	Voxel level (*P*_FWE_)	Extent (*k*)	*T*, *Z* scores	MNI coordinates (*x*, *y*, *z*) (mm)	Region
Pro-DLB vs. control subjects	<0.001	2361	7.1, 6.0	11, 57, 18	Right superior frontal
			7.1, 6.0	9, 50, 13	Right paracingulate
	<0.001	461	6.8, 5.8	−38, 11, 13	Left insula
	0.001	1321	6.4, 5.5	−29, 24, −6	Left insula
	0.001	340	6.1, 5.3	−8, −63, 45	Left precuneus
	0.002	53	5.9, 5.2	14, −43, 43	Right precuneus
	0.002	359	5.9, 5.2	30, 29, −3	Right inferior frontal
	0.006	137	5.6, 5.0	39, 5, 7	Right insula
	0.009	35	5.5, 4.9	5, 68, −3	Right medial frontal
	0.016	25	5.3, 4.8	−14, 60, 7	Left medial frontal
	0.017	15	5.3, 4.8	−29, 29, 40	Left middle frontal
	0.018	31	5.3, 4.7	26, 56, −11	Right superior frontal
	0.033	12	5.1, 4.6	−14, 47, 0	Left anterior cingulate
Pro-AD vs. control subjects	<0.001	24,640	9.8, 7.4	12, 59, −15	Right medial frontal
			8.6, 6.8	50, 47, −3	Right inferior frontal
			8.0, 6.5	−30, −19, −18	Left hippocampus
			7.4, 6.2	42, 54, −14	Right middle frontal
			7.3, 6.1	−44, 26, −5	Left inferior frontal
			7.3, 6.1	−12, 62, −3	Left medial frontal
			7.3, 6.1	−9, 69, −-9	Left superior frontal
	<0.001	1144	6.7, 5.7	35, 21, −42	Right temporal pole
			6.1, 5.3	39, 8, −33	Right superior temporal
	0.001	1881	6.3, 5.5	12, −39, 42	Right posterior cingulate
			6.1, 5.3	−8, −39, 40	Left posterior cingulate
	0.002	176	6.0, 5.2	33, −81, −53	Right posterior cerebellum
	0.003	67	5.9, 5.2	11, −9, 39	Right mid-cingulate
	0.004	392	5.8, 5.1	15, 24, 61	Right superior frontal
	0.004	94	5.8, 5.1	−47, −84, −9	Left inferior occipital
	0.004	145	5.8, 5.1	−5, −48, −27	Left anterior cerebellum
	0.005	527	5.7, 5.0	−33, −70, 39	Left precuneus
	0.007	178	5.6, 5.0	−11, 15, 39	Left mid-cingulate
	0.008	113	5.5, 4.9	48, −76, 24	Right middle temporal
	0.011	12	5.4, 4.8	18, −72, 56	Right superior parietal
	0.011	137	5.4, 4.8	29, −12, −14	Right hippocampus
	0.012	42	5.4, 4.8	−60, 2, −27	Left middle temporal
Pro-AD vs. pro-DLB	0.006	79	5.6, 4.9	20, −42, 60	Right superior parietal
Pro-DLB (hallucinators vs. non-hallucinators)	0.002^a^	82	3.1, 2.8	−5, −84, 18	Left cuneus BA17

*Abbreviations: BA* Brodmann area, *FWE* family-wise error, *MNI* Montreal Neurological Institute, *pro-AD* prodromal Alzheimer’s disease, *pro-DLB* prodromal dementia with Lewy bodies, *VBM-DARTEL* voxel-based morphometry with diffeomorphic anatomical registration through exponentiated lie algebra

Table depicts voxel-level significance (*P*_FWE_), spatial extent (*k*), *T* and *Z* scores, MNI coordinates and anatomical region

^a^Uncorrected *P* value

A more extensive atrophic pattern was apparent in pro-AD compared with HC, showing significant bilateral GM loss in the hippocampal, frontal (inferior, medial, superior gyri) and middle temporal gyrus, as well as in the posterior and mid-cingulate regions (Fig. 2b). Other unilateral areas included the cerebellum, precuneus, inferior occipital, and middle frontal and superior parietal regions. Table 2 shows the location and peak significance of the regions. No GM deficits were observed in HC which exceeded those in pro-AD.

Between patient groups, differences in GM atrophy were much decreased and confined to the right superior parietal region in pro-AD relative to pro-DLB (Fig. 2c). Table 2 presents the location and peak significance of this area. No significant GM loss was found in pro-DLB that was greater than that in pro-AD.

**Fig. 3:**
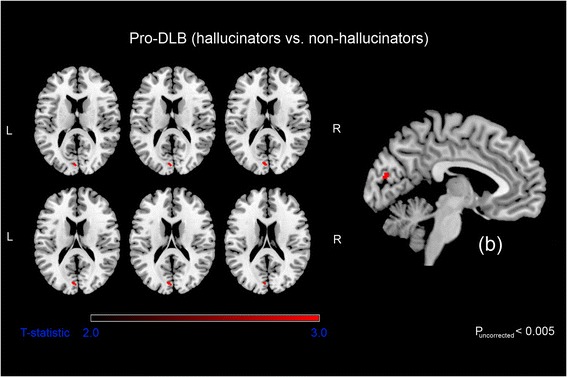
Significant grey matter loss in prodromal dementia with Lewy bodies (pro-DLB) visual hallucinators compared with pro-DLB non-hallucinators. Results (*P*_uncorrected_ ≤ 0.005) superimposed on a magnetic resonance imaging T1-weighted brain template scan in axial (on the left) and sagittal (on the right, b). *L* left, *R* right

As an exploratory exercise, we also investigated patterns of GM loss between visual hallucinators and non-hallucinators in pro-DLB. Significant GM volume loss in the left cuneus was associated with the presence of visual hallucinations (Fig. 3). Table 2 presents the location and peak significance of these areas. GM atrophy in non-hallucinators did not exceed that in hallucinators.

### Regression analyses

Effects of GM volume loss on parkinsonism and fluctuations were separately investigated in pro-DLB (age, sex and TIV adjusted). Associations between any of these measures and GM volume did not yield any significant results.

## Discussion

We report distinct GM atrophy in pro-DLB and pro-AD compared with HC. Compared with HC the group, the pro-DLB group was characterised by cortical atrophy in insulae, structures and anterior cingulate cortex. Compared with HC, pro-AD was characterised by a widespread cortical atrophic pattern which included the hippocampi and temporal, parietal and frontal structures. The comparison of patients with pro-AD with patients with pro-DLB demonstrated more atrophy the right parietal lobe in the former than in the latter group.

The results we obtained with patients with pro-AD are in accord with the literature. The progression of atrophy with time in pro-AD was previously described as beginning in the medial temporal lobes [37], then extending to the parietal and finally the frontal lobes [27, 38, 39]. On one hand, the strong involvement of the frontal lobe is unusual in MCI-AD [38]. On the other hand, more impaired MCI [40] and MCI converters to AD dementia [41] have greater atrophy in the frontal lobe at the initial scan.

The atrophy found in patients with pro-DLB compared with HC has not been described previously, to our knowledge. Notably, we obtained similar results with the same patients on the basis VBM-DARTEL as we did with FreeSurfer methods [13], showing clear atrophic involvement of the anterior insulae in pro-DLB. Previously, we observed that cortical thinning was more apparent in the right insula, whereas in the present study, we observed GM insular atrophy bilaterally. This may reflect subtleties between MRI techniques when quantifying volumetric changes [42]. However, the discovery of the insula change in pro-DLB with two techniques in the same cohort reinforces the link between pro-DLB and early changes in the insula. This overlaps with voxel-wise meta-analytic data on cortical atrophy of patients with DLB at the stage of dementia, based on findings of bilateral insular and basal ganglia atrophy [22]. The insula is involved in integrating somatosensory, autonomic and cognitive-affective information to guide behaviour [43], and specifically the anterior insula has been described as part of a ‘salience network’ due to its consistent activation during cognitively demanding tasks. It also has been implicated in switching brain networks involved in cognition, including the central executive and default mode network [44]. The anterior insula has an abundance of specific neurons, namely the von Economo neurons (VENs), located in layer 5 of the cortex with a predominance in the right hemisphere, the same region we have found to be atrophic and thinner in pro-DLB [45]. Notably, in the right anterior cingulate cortex, an area which we found to be atrophic in this study, is also rich in VENs. Because of the larger size of VENs compared with pyramid neurons, they are purported be involved in the fast assessment of complex situations [45], as well as in the salience network function [46], and thus it might be hypothesised that deficits in this region might be pertinent to the cognitive slowing and attentional deficits which characterise DLB. This is supported by the observation that increasing functional dyssynchrony between frontal areas inclusive of the insula and parietal areas, as measured using resting state MRI, is associated with increasing cognitive fluctuations in patients with DLB [24]. Whether there is a specific vulnerability of VENs in DLB remains to be confirmed, however.

When we compared pro-DLB with pro-AD, we found subtle right parietal GM volume reductions in patients with pro-AD, and this is in line with our previous findings demonstrating cortical thinning of this region in AD compared with DLB [13]. Notably, we did not find evidence of any relative atrophy of the hippocampi in pro-AD compared with pro-DLB. However, this is consistent with our visual rating data which demonstrated that both groups had relatively mild hippocampal atrophy at the prodromal stage, with only subtle differences between the two diseases. Longitudinally, however, it might be expected that there would be an increasing divergence between pro-AD and pro-DLB cohorts, with greater hippocampal loss in the former; certainly, longitudinal comparisons in established dementia demonstrate that marked temporal and hippocampal thinning is a feature of AD but not DLB [47].

We found an association between GM loss in the left cuneus and visual hallucinations in our pro-DLB group. The cuneus is a secondary visual area (Brodmann area 18) and is of importance for recognition and extraction of object features (shape, colour, movement). Dysfunction of the cuneus is responsible for errors in visual processing [48]. The involvement of this visual area in hallucinations of patients with DLB is consistent with previous studies [48–50]. The involvement of the cuneus is congruent with the top-down/bottom-up models on hallucinations [51], since we have found a key region for visual processing (bottom-up).

Our study has some limitations. In the present study, we have used a combination of pre-existing criteria for MCI and DLB, but a primary issue is that operational consensus criteria for pro-DLB remain to be established. In addition, whilst patients continue to be followed longitudinally, our cohort is relatively recent in its inception, and the definite diagnostic trajectory of patients with prodromal impairment remains to be established either clinically or neuropathologically, particularly with regard to DLB. Moreover, the possibility that an incorrect classification of patients confounded the results cannot be excluded. However, we used the McKeith et al. criteria, which have an excellent specificity (>95 %) [52, 53] compared with gold standard neuropathological diagnosis. In addition, we excluded other pathologies, such as psychiatric illness, other neurological diseases and co-occurrence of AD and DLB (see Fig. 1 flowchart). Furthermore, the majority of our patients with pro-DLB had RBD (>50 %), which enhances the specificity of the diagnosis [54]. We also systematically looked for subtle clinical symptoms such as anosmia/hyposmia, constipation and other autonomic features (data not shown) [55], as these have previously been demonstrated to improve the diagnostic specificity of patients with pro-DLB [55]. Finally, we used CSF analysis for most of the patients and obtained similar results for patients with AD and patients with DLB.

From a technical perspective, it could also be argued that a drawback of our study is the fact that data were collected from two sites with differing imaging protocols. Unfortunately, we were not able to include MRI sequences in the model, as the relative numbers in each group were disproportionately represented (i.e., 30 HC at NCL, 3 at SXB; 1 AD at NCL, 26 at SXB; and 2 DLB at NCL, 26 at SXB). Therefore, it was difficult to obtain accurate parametric estimates without considerable error. However, centre effects were less likely to have influenced the between-patient contrast, as the results did not significantly differ when we examined only SXB patients in the analysis. A single-centre study should be conducted to confirm these results. Last, in a possible future analysis using resampling/subsampling techniques, the reliability of the prodromal results could be examined for validation purposes, although this would require larger cohorts.

## Conclusions

Our data suggest that patients with prodromal disease have different patterns of atrophy depending on the pathology. Patients with pro-DLB seem to have more restricted areas of atrophy inclusive of the insulae and anterior cingulate cortex, and patients with pro-AD have more diffuse areas. New studies using MRI are acutely needed, particularly those considering perfusion, functional connectivity and structural connectivity, as well as other imaging modalities, such as positron emission tomography metabolic and amyloid radiotracers, to better understand the specificity of patients with pro-DLB.

## Abbreviations

Aβ_1–42_, amyloid-β_1–42_; AD, Alzheimer’s disease; ANOVA, analysis of variance; BA, Brodmann area; CAF, Clinician Assessment of Fluctuation; CDR, Clinical Dementia Rating; ChEI, cholinesterase inhibitor; CSF, cerebrospinal fluid; 3D, three-dimensional; DARTEL, diffeomorphic anatomical registration through exponentiated lie algebra; DLB, dementia with Lewy bodies; Dopa, levodopa or dopaminergic agonists; DSM-V, *Diagnostic and Statistical Manual of Mental Disorders, Fifth Edition*; FWE, family-wise error; GM, grey matter; HC, healthy elderly control subjects; MCI, mild cognitive impairment; MMSE, Mini Mental State Examination; MNI, Montreal Neurological Institute; MRI, magnetic resonance imaging; NCL, old age psychiatry, geriatric medicine or neurology services from Newcastle upon Tyne; NL, neuroleptic; pro-AD, prodromal Alzheimer’s disease; pro-DLB, prodromal dementia with Lewy bodies; RBD, rapid eye movement sleep behaviour disorder; SXB, tertiary memory clinic of Strasbourg; TE, echo time; TIV, total intracranial volume; TMT, Trail Making Test; TR, repetition time; UPDRS, Unified Parkinson’s Disease Rating Scale; VBM, voxel-based morphometry; VEN, von Economo neuron; WM, white matter
